# Microvascular endothelial dysfunction in skin is associated with higher risk of heart failure with preserved ejection fraction in women with type 2 diabetes: the Hoorn Diabetes Care System Cohort

**DOI:** 10.1186/s12933-023-01935-z

**Published:** 2023-09-01

**Authors:** Elisa Dal Canto, L. van Deursen, A. G. Hoek, P. J. M. Elders, H. M. den Ruijter, J. van der Velden, V. van Empel, E. H. Serné, E. C. Eringa, J. W.J. Beulens

**Affiliations:** 1 Department of Experimental Cardiology, Division Heart and Lungs, UMC Utrecht, Mathias van Geunsgebouw, room 03.03. Postbus 85500 | 3508 GA, Utrecht, The Netherlands; 2 Department of General Practice and Epidemiology, Julius Center for Health Sciences and Primary Care, University Medical Center Utrecht, Utrecht, the Netherlands; 3 Department of Epidemiology and Data Science, Amsterdam University Medical Center, location Vrije Universiteit, Amsterdam, The Netherlands; 4 Amsterdam Cardiovascular Sciences, Amsterdam, The Netherlands; 5 Department of General Practice and Elderly Care Medicine, Amsterdam University Medical Center, Amsterdam, The Netherlands; 6 Department of Physiology, Amsterdam University Medical Center, Amsterdam, The Netherlands; 7 Department of Cardiology, CARIM School for Cardiovascular Diseases, Maastricht University Medical Center, Maastricht, The Netherlands; 8 Department of Vascular Medicine & Diabetes Center, Amsterdam University Medical Center, location Vrije Universiteit, Amsterdam, The Netherlands; 9 Department of Physiology, CARIM School for Cardiovascular Diseases, Maastricht, The Netherlands; 10 Julius Center for Health Sciences and Primary Care, University Medical Center Utrecht, Utrecht, the Netherlands; 11 Amsterdam Public Health, Amsterdam, The Netherlands

**Keywords:** Type 2 diabetes, Microvascular function, Diastolic dysfunction, Heart failure with preserved ejection fraction, Sex differences, Cardiovascular prevention

## Abstract

**Background:**

Microvascular dysfunction plays a crucial role in complications of type 2 diabetes and might contribute to heart failure with preserved ejection fraction (HFpEF), a disease that disproportionally affects women. We aimed to investigate if presence and degree of microvascular dysfunction (MVD) in skin relates to markers of left ventricular diastolic dysfunction (LVDD) and HFpEF risk in adults with type 2 diabetes, and whether sex modifies this association.

**Methods:**

We recruited 154 participants (50% women) from the Hoorn Diabetes Care System Cohort, a prospective cohort study, for in vivo evaluation of skin MVD, echocardiography and blood sampling. MVD was assessed by laser speckle contrast analysis combined with iontophoresis of insulin, acetylcholine and sodium nitroprusside (SNP). We performed a cross-sectional analysis of the association between perfusion responses and echocardiographic and clinical markers of LVDD and the H2FPEF score by multivariable linear regression analysis adjusted for confounders. Sex was evaluated as a potential effect modifier and the analysis was stratified.

**Results:**

Mean age was 67 ± 6y, mean HbA1c 7.6 ± 1.3%. Women were more frequently obese (54.5 vs. 35.1%), had higher NT-proBNP plasma levels (80, IQR:34–165 vs. 46, 27–117 pg/ml) and E/E’(13.3 ± 4.3 vs. 11.4 ± 3.0) than men. Eleven women and three men were diagnosed with HFpEF, and showed lower perfusion response to insulin than those without HFpEF. A lower perfusion response to insulin and acetylcholine was associated with higher HFpEF risk in women, but not men (10% decreased perfusion response was associated with 5.8% [95%CI: 2.3;9.4%] and 5.9% [1.7;10.1%] increase of the H2FPEF score, respectively). A lower perfusion response to SNP was associated with higher pulmonary arterial systolic pressure in men while a lower perfusion response to acetylcholine associated with higher LV mass index in women and with worse LV longitudinal strain in the total population. No significant associations were found between perfusion responses and conventional LVDD markers.

**Conclusions:**

Impaired microvascular responses to insulin and acetylcholine in skin confers a higher risk of HFpEF in women with type 2 diabetes. In vivo measures of systemic MVD could represent novel risk markers for HFpEF, opening new avenues for the prevention of HFpEF in type 2 diabetes.

**Supplementary Information:**

The online version contains supplementary material available at 10.1186/s12933-023-01935-z.

## Introduction

Heart failure with preserved ejection fraction (HFpEF) is the predominant HF phenotype among people with diabetes, and its prevalence is increasing [1, 2]. Despite this alarming trend, the diagnosis of HFpEF remains challenging. According to current guidelines, the diagnosis of HFpEF should include the presence of symptoms and signs of HF, a left ventricular ejection fraction (LVEF) ≥ 50% and the evidence of cardiac abnormalities suggestive of LV diastolic dysfunction (LVDD), including increased natriuretic peptides [3]. However, this approach is complicated by the fact that symptoms are often mild or absent, and natriuretic peptides can be in the normal range especially in obese patients. Accordingly, important issues involving HFpEF remain unaddressed, including the existence of an early HFpEF stage, when risk factors are present but symptoms are not yet manifest and cardiac abnormalities and LVDD might still be reversible [4]. Recently, the H2FPEF-score has been proposed to aid the diagnosis in symptomatic euvolemic patients [5]. This score enables discrimination between HFpEF and non-cardiac causes of dyspnea and relates to both adverse outcomes in HFpEF patients [6], and to future HF in stable outpatients with cardiovascular risk factors [7].

Microvascular inflammation and dysfunction (MVD), present either systemically or in the coronary circulation are common in type 2 diabetes (T2D), and associate to HFpEF development [8, 9]. Indeed, systemic inflammation, resistance to insulin’s physiological actions, altered myocardial energetics and MVD are highly implicated in the pathogenesis of HFpEF in patients with obesity, T2D and metabolic syndrome [9, 10]. Cardiac endothelial MVD may then induce cardiomyocyte hypertrophy and stiffness and favor interstitial fibrosis by reducing nitric oxide (NO)-cyclic guanosine monophosphate (cGMP) signaling, thereby contributing to LVDD and HFpEF [14–16]. Both endothelium-dependent and endothelium-independent mechanisms are responsible of MVD in HFpEF and T2D [11]. On the other hand, whether and to what extent systemic and coronary MVD in HFpEF are related is uncertain. Furthermore, the involvement of systemic MVD in people with various degrees of unexplained dyspnea and higher HFpEF risk is unclear. Nowadays several techniques can be used to investigate systemic MVD, among which forearm and finger plethysmography, arterial tonometry and laser-Doppler flowmetry are the most widely used [12]. To this regard, it is important to point out that different microvascular techniques and stimuli provide distinct functional information on the status of the microcirculation.

Women are twice as likely to develop HFpEF than men and their predisposition to non-obstructive microvascular angina and endothelial MVD is likely to play a crucial role [13, 14]. Importantly, the presence of diabetes increases HF risk of 5-fold in women and 2.4-fold in men [15, 16]. In addition, women with diabetes display a worse cardiac phenotype with higher prevalence of concentric remodeling compared to men [13]. Besides diabetes, obesity is another cardiovascular risk factor that more strongly contributes to the development of HFpEF in women compared to men [13]. The epidemiologic associations of diabetes and obesity with HFpEF in women might suggest that insulin resistance and resistance to insulin’s microvascular effects may contribute to the sex differences in HF. Indeed, insulin resistance, impaired insulin signaling and obesity associate with more severe adipose tissue dysfunction, inflammation, MVD and decreases in cardiac efficiency in women than men [17, 18]. Because MVD precedes microvascular complications and cardiovascular diseases such as HFpEF in people with T2D [19], investigation of the relationship between multiple mechanisms of MVD and HFpEF in men and women with diabetes can help to identify those at increased risk of developing this disease.

In the present study we characterized systemic MVD in male and female patients with T2D, and investigated its association with clinical and echocardiographic markers of LVDD/HFpEF with special emphasis to sex differences. We hypothesized that a decreased microvascular function is associated with a higher HFpEF risk, especially in women.

## Methods

### Study population

The present study was performed in the Hoorn Diabetes Care System cohort (DCS), which consists of > 13,000 people with T2D in the region of West Friesland in the Netherlands. Participants underwent annual check-ups of T2D risk factors and microvascular complications. The presence of T2D was established if one of the following was reported by a general practitioner: (1) one or more symptoms combined with elevated fasting plasma glucose of ≥7.0 mmol/L (126 mg/dL) or elevated random plasma glucose of ≥11.1 mmol/L (200 mg/dL); (2) in the absence of symptoms, at least two elevated plasma glucose concentrations on two different occasions [20]. From November 2019 onwards, 848 men and women from the DCS cohort were invited to participate in a cardiovascular screening for the early detection of LVDD and HFpEF. People aged 50–75 years old with T2D for at least one year were considered eligible and those with advanced diabetes complications, valvular heart disease or cardiomyopathies were excluded. Participants were assessed for the presence of symptoms and signs related to HF through a questionnaire that was adapted from the Kansas City Cardiomyopathy Questionnaire, and underwent transthoracic echocardiogram, blood sampling for natriuretic peptides, assessment of arterial stiffness (by measurement of carotid-femoral pulse wave velocity and augmentation index with SphygmoCor technology and ankle-brachial index), blood pressure measurements and a CT-scan of the legs and heart to quantify vascular calcification levels and patterns. A subgroup of 154 participants, 77 men and 77 women, underwent additional investigation of skin MVD, performed by laser speckle contrast analysis (LASCA) combined with iontophoresis. Three participants were excluded (one from analysis on the first LASCA protocol, and two from analysis on the second) because of unsuccessful measurements (leakage of the drugs out of the drug chambers or loss of contact between iontophoresis chambers and electrodes).

### Assessment of microvascular function

Microvascular function was evaluated in the skin using semi-quantitative LASCA technology of the PeriCam PSI system (Perimed instruments, Järfälla-Stockholm, Sweden), a novel non-invasive real-time imaging technique that measures perfusion of blood cells, mainly erythrocytes, in the skin microvasculature. It can be used in combination with iontophoresis of various vasoactive substances to evaluate endothelium-dependent and -independent mechanisms of MVD. Iontophoresis is a method for non-invasive transdermal delivery of vasoactive agents to the cutaneous microcirculation based on the net movement of ions using a low-intensity electrical current [21]. Microvascular endothelium-dependent vasodilator function was evaluated using iontophoresis of acetylcholine (1%) and insulin (1%). Microvascular endothelium-independent vasodilator function was evaluated using iontophoresis of sodium nitroprusside (SNP, 0.1%) [22]. The tests were performed after an acclimatization period of approximately 10 min in a temperature-controlled room (22 °C). The participants were in supine position to minimize the effect of body position on microvascular circulation. Skin perfusion was measured on the forearm, the skin penetration depth of LASCA is about 300 μm [22]. Dedicated software (PimSoft version 1.6) was used to determine the baseline perfusion, the perfusion plateau induced by the delivery of each substance and to calculate the maximum absolute and relative change in perfusion due to the substance calculated as 100% x ([Perfusion plateau – Baseline flow]/Baseline flow). Skin perfusion outside the area of interest was used as control. A more detailed description of the procedure is presented in **Appendix A, Additional Material**.

### Assessment of cardiac function and structure

Echocardiography was performed with a Philips Affiniti 70 system by an experienced sonographer according to a specific protocol involving 2-Dimensional (2D), M-mode, Doppler, tissue Doppler and 2D speckle tracking imaging. Cardiac function and structure measures were then analyzed offline by a single trained investigator blinded to clinical information on a dedicated software (Q station, version 3.3). Among the 154 included participants, 107 underwent echocardiography on the same day as LASCA measurements and 47 participants underwent echocardiography prior to LASCA (of whom 39 underwent echocardiography 12–17 months earlier and eight < 6 months earlier), due to delays during COVID-19 restrictions.

LV diastolic function was evaluated by a set of echocardiographic parameters according to current recommendation [23]. Risk of HFpEF was assessed by the H2FPEF score [3], a validated diagnostic tool that estimates the likelihood of HFpEF. For the present study the H2FPEF score was evaluated on a continuous scale, by the following formula, resulting in a probability of HFpEF in percentages [24, 25]: *Probability of HFpEF*: (Z/(Z + 1)) x 100, where Z = e^Y^, and Y = 9.1917 + 0.0451*age + 0.1307*body mass index (BMI) + 0.0859*E/e’ ratio + 0.0520*estimated pulmonary arterial pressure (PASP) + 1.6997*atrial fibrillation (AF) (where Yes = 1, No = 0). Associations with the total score and its single components were determined, to evaluate which clinical and/or echocardiographic markers might be driving the associations. Additional echocardiographic markers of LV diastolic function (Mean E’ velocity, LV mass index [LVMI], left atrial volume index [LAVI], LV global longitudinal strain [GLS] by speckle-contrast analysis), of left atrial (LA) function (LA global longitudinal strain), and right ventricular (RV) function (tricuspid annular plane systolic excursion [TAPSE]) were assessed according to current recommendation and used singularly as additional outcome variables [26]. Finally, based on the evidence of diastolic function and/or cardiac structural abnormalities, on NT-proBNP plasma levels and on the presence of signs and symptoms of HF, a diagnosis of HFpEF was formulated [3].

### Covariates

A blood sample for the assessment of NT-proBNP plasma levels was collected on the day of the visit. Information on medical history, other biomarkers and medication use were collected during the annual diabetes check for the DCS cohort and extracted from the medical records for the purpose of this study. Fasting plasma glucose, glycated hemoglobin (HbA1c), plasma total, high-density, low-density lipoprotein cholesterol (LDL-c) and serum creatinine concentrations were as well determined during the last annual diabetes check (performed in 2020) as described elsewhere [27]. Information on diabetes duration, educational level and smoking status was obtained by self-report. High educational level was defined as higher vocational education or university, medium level as secondary education, and low level as elementary school, lower vocational training or less. BMI was calculated by dividing body weight (kg) by height (m) squared as assessed during the study visit (kg/m^2^). Systolic and diastolic blood pressure (SBP and DBP) were measured during the study visit according to a standardized protocol and arterial hypertension was defined as SBP ≥ 140 mmHg, DBP ≥ 90 mmHg and/or use of anti-hypertensive medication. Finally, during the study visit and before the assessment of microvascular function participants were asked about the time (hh:mm) since their last meal. NT-proBNP and SBP were used as additional outcome variables and related to perfusion responses.

### Statistical analyses

Baseline characteristics were presented as means and standard deviations for continuous normally distributed variables, as median and interquartile range for non-normally distributed continuous variables and as count and percentages for categorical variables. Characteristics were presented separately for men and women and independent-samples T-test, Wilcoxon Rank Sum test and Chi-Square test were used to assess sex differences in baseline characteristics depending on the distribution of the variable. Hierarchical multiple linear regression was used to assess the cross-sectional relationship between log-transformed perfusion responses (relative change in perfusion) to insulin, acetylcholine, and SNP and the H2FPEF score (including the total score and each single component) as well as additional echocardiographic and clinical markers of LVDD. A set of predefined models were fitted. A minimally adjusted model included sex, HbA1C (%), LDL-cholesterol (mmol/L), presence of hypertension (yes/no), serum creatinine (mg/dL), albumin-creatinine ratio (mg/mmol), diabetes duration (years), smoking status (never/former/current), prior cardiovascular disease (yes/no), NT-proBNP value (pg/ml), relative change in perfusion of the skin outside the measurement areas, time since last meal (hh:mm, for insulin protocol only), perfusion response to NaCl (for insulin protocol only) (model 1). Model 2 additionally adjusted for use of metformin (yes/no), insulin (yes/no) and of any anti-hypertensive medication (yes/no). The regression models investigating single outcome variables also included age (years) and BMI (kg/m^2^). Sex was assessed as an effect modifier by including interaction terms with the perfusion responses for each substance. Interaction terms were included in the fully adjusted models along with the main effects. Wald tests were used to assess whether the models with the interaction terms differed from those without. A p-value < 0.10 for the interaction was considered statistically significant. Nevertheless, results were presented separately for men and women, since this was included as such in the design of the study. Sensitivity analyses were carried out by excluding: (1) participants for whom a perfusion plateau was not clearly observed during the recording of the insulin protocol (n = 23); (2) participants who underwent MVD assessment more than six months after echocardiography (n = 39); (3) participants without any symptoms or signs suggestive of HF (NYHA class < II and/or absence of edema, n = 107). Missing values were present in a proportion ranging from 0.6% for HbA1c (%) to 16.9% for use of general antihypertensive medication and were imputed using multiple imputation, assuming that the data were missing at random. We generalized 10 imputed datasets (50 iterations) and used Rubin rules to combine the estimates of the parameters. All analyses were performed using SPSS Statistics, version 22.0 (IBM Corp, IBM SPSS Statistics for Windows, Armonk, NY, USA) and R-Studio version 3.4.2 (R Foundation for Statistical Computing, Austria). A P-value < 0.05 was considered statistically significant.

## Results

### Baseline characteristics of study participants

The population consisted of 77 men and 77 women. Women had a significantly higher plasma concentrations of NT-proBNP and LDL-c and were more often obese than men. A proportion of 21.5% of men and of 36.8% women (woman vs. men p > 0.05) showed presence of HF symptoms (dyspnea NYHA class > II). Markers of diabetes control, kidney function measures, BMI, blood pressure, cardiovascular comorbidities and medication use did not significantly differ between men and women (Table 1).

**Table 1:** Baseline demographic, clinical characteristics and medication use of study participants, stratified by sex

		Men, n = 77		Women n = 77	P value
Demographics					
Age, years		66 ± 6		67 ± 6	0.479
High education, n (%)		16 (20.8)		19 (25.3)	0.940
Current smokers, n (%)	5 (6.5)			7 (9.5)	0.827
Diabetes control					
Diabetes duration, years	14.7 (11.7–18.4)			14.6 (11.8–18.8)	0.845
HbA1C, %	7.3 (6.8–8.2)			7.3 (6.6–8.4)	0.818
Fasting plasma glucose, mmol/L	9.3 ± 2.2			9.0 ± 2.1	0.377
Symptoms, n (%)					
NYHA classes					0.136
class I	55 (78.6)			43 (63.2)	
class II	13 (18.6)			21 (30.9)	
class III/IV	2 (2.9)			4 (5.9)	
Orthopnea (> 1x / week)	2 (2.6)			4 (5.2)	0.694
Edema	2 (2.6)			4 (5.2)	0.405
Biomarkers and comorbidities					
NTproBNP, pg/ml	46.0 (26.8-116.9)			80.1 (34.0-164.8)	**0.022**
eGFR, mL/min/1.73m^2^	86.0 (75.7–98.8)			81.5 (69.1–95.0)	0.060
Albumin-creatinine ratio, mg/mmol	0.6 (0.4–1.6)			0.7 (0.5–1.4)	0.608
LDL-cholesterol, mmol/L	1.9 (1.4–2.5)			2.2 (1.6–3.2)	**0.010**
BMI, kg/m2	29.2 ± 4.3			30.6 ± 5.9	0.082
Obesity, n (%)	27 (35.1)			42 (54.5)	**0.015**
Hypertension, n (%)	40 (51.9)			37 (48.1)	0.629
Systolic blood pressure, mmHg	141 ± 17			141 ± 16	0.818
Diastolic blood pressure, mmHg	80 ± 11			77 ± 9	0.075
Atrial fibrillation, n (%)	2 (2.6)			2 (2.6)	1.000
Prior CVD, n (%)	17 (22.1)			9 (11.7)	0.085
Medication use, n (%)					
Diabetes medication					
Metformin			55 (78.6)	57 (76.0)	0.712
Sulfonylureas			31 (44.3)	29 (38.7)	0.492
DPP-4			2 (2.6)	1 (1.3)	0.560
SGLT-2 inhibitors			0 (0.0)	0 (0.0)	-
GLP-1 agonists			1 (1.4)	0 (0.0)	0.299
Insulin			25 (35.7)	33 (44.0)	0.309
Use of anti-hypertensive medication					
ACE-inhibitors/ARBs			31 (44.3)	34 (45.3)	0.899
Beta-blockers			17 (24.3)	25 (33.3)	0.230
Calcium channel blockers			12 (17.1)	17 (22.7)	0.406
Statins			48 (68.6)	48 (64.0)	0.561
Diuretics			15 (21.4)	20 (26.7)	0.461

Results are presented as mean ± standard deviation, median (interquartile range) or frequencies (percentages) and compared between men and women using t-test, Wilcoxon Rank Sum test and chi-square tests, where appropriate

HbA1c = Hemoglobin A1c. NYHA = New York Heart Association. eGFR = estimated glomerular filtration rate. LDL = low-density lipoprotein. BMI = body mass index. CVD = cardiovascular disease. DDP-4 = dipeptidyl peptidase-4 inhibitor. SGLT-2 = sodium-glucose linked transporter. GLP = Glucagon-like peptide. ACE = angiotensin converting enzyme. ARB = angiotensin receptor blocker

**Fig. 1:**
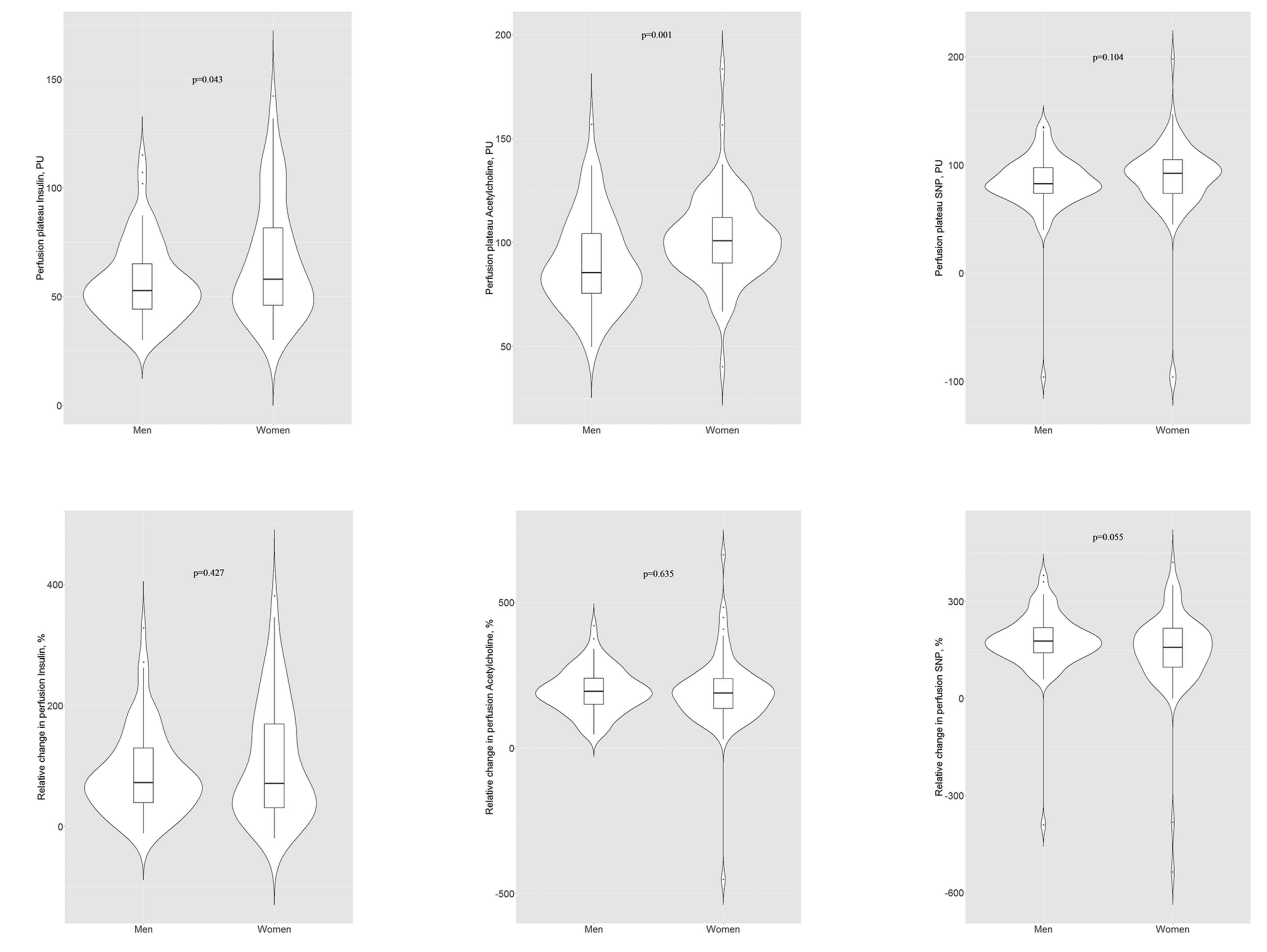
Visual summary of main microvascular function measures stratified by sex

**Fig. 2:**
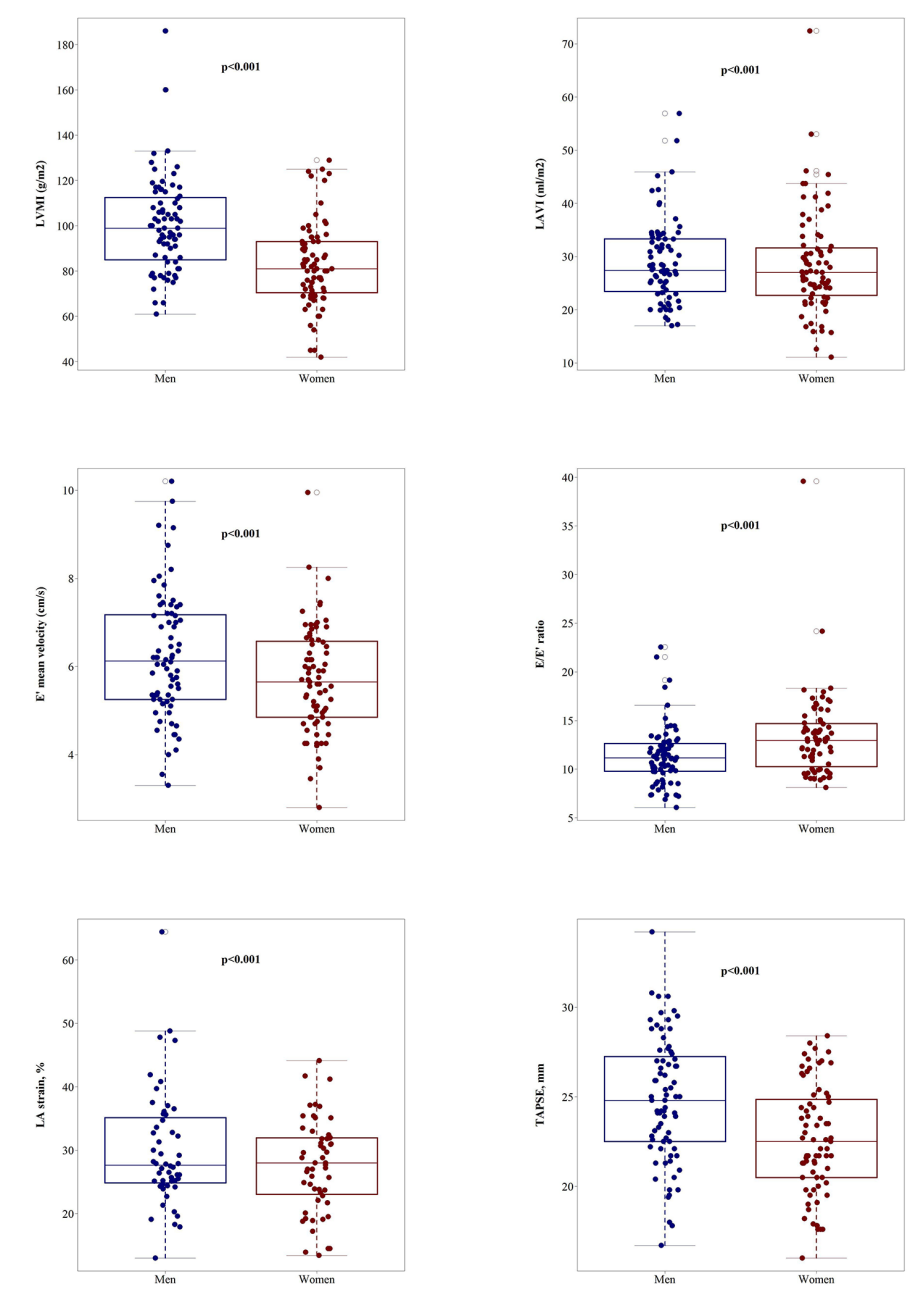
Visual summary of main echocardiographic markers of left ventricular diastolic function, left ventricular and left atrial structure, left atrial function and right ventricular function stratified by sex

With respect to microvascular function, baseline perfusion (average of baseline perfusion before each protocol), the maximal perfusion plateau during acetylcholine-stimulated perfusion and time-to-plateau during insulin-stimulated perfusion were significantly higher in women than men. However, absolute and relative changes in perfusion induced by each substance did not differ by sex (Fig. 1 and **Additional** Table 1), apart from a higher variation in responses present in women. Women showed worse LV diastolic function than men, evident from lower E’ velocities and higher E/E’ ratio. They also showed smaller LV volumes, lower stroke volume, worse left atrial function, evident from lower A’ and LA strain, smaller RV area and right atrial volume and worse RV function, evident from lower TAPSE and RV S’. Furthermore, the H2FPEF score was significantly higher in women than men (Fig. 2 and **Additional** Table 2). Based on the presence of echocardiographic findings indicative of LVDD and of symptoms and signs suggestive of HF [3], eleven women and three men were eventually diagnosed with HFpEF. Participants with HFpEF had higher BMI, lower eGFR, worse cardiac function and structure and a lower perfusion plateau and response to insulin compared to those without HFpEF (Table 2).

**Table 2:** Clinical, cardiac and microvascular function characteristics of participants diagnosed with HFpEF compared to those with no HFpEF

	Diagnosed with HFpEF (n = 14)	Not diagnosed with HFpEF (n = 140)	P-value
**Clinical characteristics**			
Age, years	69 ± 5.8	66.3 ± 6.2	0.122
Female, %	11 (79)	66 (47.1)	0.019
HbA1c, %	7.9 ± 1.5	7.5 ± 1.3	0.452
NT-proBNP pg/mL	222.0 (171.8-313.5)	54.5 (27.6-118.7)	**0.018**
eGFR,, mL/min/1.73 m2	68.6 (59.8–89.9)	86.0 (72.6–97.6)	**< 0.001**
BMI, kg/m2	35.3 ± 6.2	29.3 ± 4.8	**0.003**
**Echocardiography**			
E’ mean, cm/s	5.5 ± 1.4	6.0 ± 1.3	0.256
E/E’, mean	17.6 ± 7.4	11.8 ± 2.8	**0.012**
LVMI, g/m2	104.5 ± 20.7	91.0 ± 21.3	**0.034**
LAVI, ml/m2	37.2 ± 8.5	27.9 ± 8.3	**0.001**
PASP, mmHg	36.0 ± 4.3	29.0 ± 5.6	**0.004**
LV strain, %	-15.3 ± 1.3	-17.11 ± 2.2	**0.001**
LA strain, %	-23.4 ± 8.0	-28.6 ± 7.0	0.063
TAPSE, mm	24.2 ± 3.6	23.7 ± 3.4	0.651
**Microvascular function, perfusion responses**			
Average baseline perfusion, PU	33.8 (29.7–39.7)	33.8 (24.4–43.9)	0.968
**Insulin**			
Perfusion plateau, PU	57.0 (45.6–75.1)	46.0 (35.2–50.8)	**0.003**
Absolute change, %	12.2 ± 20.6	30.2 ± 23.7	**0.007**
Relative change, %	45.6 ± 69.2	102.6 ± 84.6	**0.011**
**Acetylcholine**			
Perfusion plateau, PU	95.3 (83.0-110.8)	86.7 (78.0-97.5)	0.152
Absolute change, %	53.6 ± 15.5	61.7 ± 26.5	0.112
Relative change, %	172.2 ± 89.5	196.1 ± 106.3	0.379
**SNP**			
Perfusion plateau, PU	88.6 (74.6-100.5)	77.8 (64.2-100.2)	0.363
Absolute change, %	47.9 ± 23.7	51.1 ± 33.6	0.660
Relative change, %	154.2 ± 84.3	162.2 ± 118.8	0.757

Results are presented as mean ± standard deviation, median (interquartile range) or frequencies (percentages) and compared between men and women using t-test, Wilcoxon Rank Sum test and chi-square tests, where appropriate

BMI = body mass index. HbA1c = Hemoglobin A1c. eGFR = estimated glomerular filtration rate. SNP = sodium nitroprusside

### Measures of microvascular function and risk of HFpEF

We hypothesized decreased microvascular function to be associated with higher HFpEF risk. We observed significant associations in both the whole population and separately in men and women (Table 3). In particular, a higher perfusion response to insulin was associated with a lower H2FPEF score (B= -38.8, 95% confidence interval (CI): -62.9;-14.6). Although no significant effect modification by sex was observed (p-interaction = 0.296), each 10% reduction in the perfusion response to insulin was associated with 5.8% increase in the H2FPEF score in women (B= -58.1, 95% CI: -93.6;-22.7), but not in men (B = 13.3, 95% CI: -48.0;21.5). The association between perfusion response to insulin and the H2FPEF score was driven by age in both sexes and by BMI in women. A higher perfusion response to acetylcholine was also associated with a lower H2FPEF score (B= -27.4, 95% CI: -52.2; -2.5). Although a significant effect modification by sex was not observed (p-interaction = 0.642), each 10% reduction in the perfusion response to acetylcholine was associated with 5.9% higher H2FPEF score in women (B= -59.2, 95% CI: -101.2; -17.2), but not in men. The association with the H2FPEF score was in this case driven by age in men and by BMI in women. Although a significant association was found between perfusion response to SNP and H2FPEF score for model 1, the association was no longer significant in the fully adjusted model. Perfusion response to SNP was associated weakly with age (in the total population) and PASP (in men) (model 2, **Table 4**).

**Table 3:** Associations between log-transformed perfusion response for each substance, the H2FPEF score and the single components of the H2FPEF score in the total study population and in men and women separately

	Total population B (95% CI)	Men B (95% CI)	Women B (95% CI)
Perfusion response to Insulin		N = 75	N = 74
H2FPEF score			
Model 1	-42.5 (-66.9; -18.1)**	-19.9 (-55.5; 15.7)	-60.4 (-93.9; -26.9**
Model 2	-38.6 (-62.9; -14.3)**	-14.3 (-49.3; 20.6)	-60.3 (-95.0; -25.6)**
Age	-16.2 (-25.1; -7.2)**	-18.1 (-33.3; -2.8)*	-14.7 (-26.2; -3.2)*
BMI	-11.0 (-18.8; -3.2)**	-6.0 (-17.1; 5.1)	-13.9 (-26.4; -1.3)*
AF	0.1 (-0.1; 0.3)	0.2 (-0.1; 0.5)	0.0 (-0.2; 0.2)
E/E’	-2.0 (-8.0; 4.1)	1.4 (-6.0; 8.8)	-5.7 (-15.6; 4.3)
PASP	-5.5 (-15.3; 4.2)	-6.2 (-23.8; 11.4)	-7.4 (-21.1; 6.3)
Perfusion response to Acetylcholine		N = 74	N = 75
H2FPEF score			
Model 1	-26.5 (-50.9; -2.1)*	-14.5 (-50.1; 21.1)	-53.6 (-94.4; -12.8)*
Model 2	-26.3 (-51.5; -1.0)*	-9.0 (-45.9; 27.8)	-57.4 (-100.2; -14.6)**
Age	-11.5 (-20.7; -2.2)*	-24.5 (-39.7; -9.4)**	-6.4 (-20.7; 7.9)
BMI	-6.4 (-14.3; 1.6)	-0.1 (-12.3; 12.1)	-17.5 (-31.2; -3.7)*
AF	-0.0 (-0.2; 0.1)	-0.1 (-0.4; 0.2)	0.1 (-0.1; 0.4)
E/E’	-0.3 (-6.2; 5.6)	2.8 (-5.2; 10.7)	-2.8 (-13.8; 8.1)
PASP	-7.2 (-16.9; 2.6)	-6.1 (-24.6; 12.5)	-12.1 (-26.6; 2.4)
Perfusion response to SNP		N = 74	N = 75
H2FPEF score			
Model 1	-7.6 (-14.6; -0.7)*	-38.1 (-78.7; 2.5)	-8.1 (-17.1; 1.0)
Model 2	-6.2 (-13.1; 0.7)	-26.0 (-67.9; 16.0)	-6.4 (-15.7; 2.9)
Age	-3.0 (-5.4; -0.6)*	-16.4 (-35.4; 2.5)	-1.9 (-4.8; 1.0)
BMI	-1.1 (-3.3; 1.1)	-5.5 (-18.8; 7.8)	-1.1 (-4.2; 1.9)
AF	-0.0 (-0.1; 0.0)	0.0 (-0.3; 0.4)	0.0 (-0.0; 0.1)
E/E’	-0.0 (-1.6; 1.6)	4.2 (-4.6; 12.9)	-0.2 (-2.5; 2.1)
PASP	-1.0 (-3.7; 1.8)	-24.2 (-43.9; -4.6)*	-0.8 (-4.3; 2.7)

The determinants (relative change in perfusion from baseline to plateau for each of the substances) were first log-transformed and then added to the model

* P < 0.05 .** p < 0.01. BMI: body mass index. AF: atrial fibrillation. PASP: pulmonary arterial systolic pressure. SNP = sodium nitroprusside

^1^The continuous H2FPEF score is an estimation of the probability of developing HFpEF, based on the formula developed by Reddy et al. [24]

Model 1 adjusts for sex, HbA1c, LDL cholesterol, presence of hypertension, serum creatinine, diabetes duration, smoking status, prior CVD, NTproBNP value, and change in perfusion of the skin outside of the measurement area. For the regression analyses with relative change in perfusion due to insulin, model 1 additionally adjusts for change in perfusion due to delivery of NaCl and time since last meal. Model 2 additionally adjusts for metformin use, insulin use, use of anti-hypertensive medication

P-interactions with sex: relative change insulin: 0.296; relative change Acetylcholine: 0.642; relative change SNP: 0.508

### Measures of microvascular function and markers of LVDD and HFpEF

We hypothesized that measures of MVD might be also associated with other relevant echocardiographic and clinical markers of LVDD and HFpEF. Indeed we observed a significant negative association between perfusion response to acetylcholine and LVMI in women (B= -59.5, 95% CI: -104.3; -14.6) and a positive association between perfusion response to acetylcholine and LVGLS in the total population (B = 3.3, 95% CI: 0.0; 6.6) (model 2, **Additional** Table 3). Conversely, we did not observe any significant associations between measures of MVD and traditional echocardiographic markers of LVDD such as E’ velocity, E/E’ or LAVI.

### Sensitivity analyses

Sensitivity analyses excluding participants without apparent insulin perfusion plateau and those who underwent MVD assessment more than six months after echocardiography did not significantly change the results of the associations between perfusion responses to insulin and the H2FPEF score, attenuated the association between perfusion response to acetylcholine and the H2FEPF score (albeit the effect sizes were similar) and overall attenuated the associations between perfusion responses and single components of the H2FPEF score, except for the association between perfusion response to SNP and PASP in men. For the additional echocardiographic markers, the associations between perfusion response to insulin and LVMI and between acetylcholine and LVGLS were no longer significant in sensitivity analyses. Conversely, sensitivity analyses yielded positive associations between perfusion response to insulin and LVMI in the general population and in women and between perfusion response to insulin and TAPSE. Sensitivity analysis performed only in participants with signs and/or symptoms of HF (n = 47) did not yield any significant association, most likely because of limited statistical power (**Additional Table 5**). Finally, we checked whether perfusion responses to insulin and acetylcholine might be associated with each other, considering both measure features of endothelial-dependent vasodilation. These responses were associated in the crude analysis (r = 0.273, p = 0.001) but not in the adjusted one, their correlation being driven by cardiovascular risk factors.

## Discussion

In this study, we found that MVD in skin was significantly associated with higher risk of HFpEF, particularly in women with T2D. Women displayed a worse diastolic function than men, while vasodilator responses did not differ significantly between the sexes. A higher proportion of women were eventually diagnosed with HFpEF (11 women vs three men). Participants with HFpEF had worse cardiac function and structure and a lower perfusion response to insulin compared to those without HFpEF. We observed that decreased perfusion responses to insulin and to acetylcholine were associated with higher risk of HFpEF in the total population, independently of common cardiovascular risk factors and cardiovascular pharmacotherapy use. However, after stratifying by sex these associations were only present among women and not men. Age and especially BMI were the components of the H2FPEF score driving these associations in women, while a higher perfusion response to SNP was associated with a lower PASP in men. Lastly, a higher perfusion response to acetylcholine was associated with a lower LVMI and a higher LVGLS, in women and in the total population respectively. However, we did not observe any significant association between MVD measures and most relevant LVDD markers, such as E/E’.

### Sex differences in microvascular dysfunction in diabetes and *HFpEF*

A growing body of evidence points towards MVD as a key pathophysiological mechanism in HFpEF [11, 28–30]. Since women are more susceptible to microvascular angina and microvascular endothelial dysfunction [13, 31], MVD might be more important for HFpEF in women than men. Interestingly, different proteomic correlates of MVD were found in men and women with HFpEF [32], suggesting sex-specific drivers of MVD in HFpEF. Nonetheless, in our study we did not observe differences in microvascular function responses between the sexes. Previous studies investigating MVD in HFpEF reported a similar prevalence for both sexes [11, 33]. However, these findings cannot be directly compared with those of the present analysis because of the different study populations of people with HFpEF versus T2D at-risk subjects in the present study. Results from studies in T2D are on the other hand inconsistent in terms of sex differences [34, 35]. Such inconsistencies relate to multiple factors, such as the vascular bed investigated, microvascular technique and stimuli used, and characteristics of the study population. In our study, women showed higher baseline perfusion and perfusion plateaus than men, while perfusion responses did not differ between the sexes. While comparing microvascular parameters and defining MVD in men and women, we should also point out that evaluating them on the same scale might give bias, as ranges of normality for microvascular function may differ between the sexes. Vascular and cardiac function and structure differ between men and women [36] and sex-specific cut-offs already exist for LVMI for instance [36], as women generally have smaller hearts and a lower cardiac output than men. It has been suggested that sex-specific cut-offs should also be used for LVEF, since women tend to display higher LVEF than men, or even measures of LVDD [9, 28], and might also be needed for microvascular parameters. However, current guidelines do not encompass sex differences nor recommend sex-specific diagnostic criteria. The present study confirmed such differences, with women displaying smaller LVMI and volumes, an overall worse LV diastolic function and a higher prevalence of HFpEF.

### Systemic microvascular dysfunction and HFpEF risk

We found a significantly lower perfusion response to insulin in participants with HFpEF compared to those without HFpEF. Furthermore, we observed a significant and independent association between peripheral microvascular responses to insulin and acetylcholine and the H2FPEF score. Age and BMI were mostly responsible of the associations. Vasodilator actions of insulin are mediated by activation of NO synthase (eNOS) via phosphorylation by Akt and subsequent production of NO [37]. The link between impaired insulin actions and obesity is well-established: insulin resistance develops with increasing BMI and advanced age, particularly in women [10, 38]. Obesity is one of the strongest risk factors for HFpEF development, and drives a specific cardio-metabolic, inflammatory phenotype which is increasingly prevalent, characterized by altered myocardial energetics and higher disease severity [10, 39]. Our findings suggest that impaired microvascular effects of insulin, i.e. microvascular insulin resistance, might be an important driver of HFpEF in older obese women with T2D. BMI was also responsible of the association between perfusion response to acetylcholine and the H2FPEF score. Vasodilator effects of acetylcholine rely on calcium-dependent eNOS activation, through binding of acetylcholine to receptors on the endothelial cell membrane and consequent increase of the intracellular concentration of calcium, which binds to calmodulin, leading to eNOS activation and NO production. Obesity drives MVD through decreased levels of adiponectin and increased levels of free fatty acids, with consequent inflammation and endothelial dysfunction [10]. Our findings suggest a possible involvement of altered calcium handling and signaling and impaired eNOS activation in the development of HFpEF in women. Altogether, these observations suggest that a generalized impairment in eNOS activation could represent a mechanism of HFpEF risk in women. This is also in line with recent observations in engineered heart tissue that endothelial cells control cardiomyocyte function, where the endothelium-derived endothelin plays a crucial role in cardiomyocyte dysfunction [40].

No association was observed between systemic microvascular response to SNP and HFpEF risk. SNP is an endothelium-independent relaxing agent and acts directly on the vascular smooth muscle cells, by increasing cGMP via release of NO. However, a significant association was observed between perfusion response to SNP and PASP as a single component of the score, in men but not women, possibly indicating a sex-specific role for impaired endothelium-independent MVD in the pathophysiology of PH in men.

Even though established risk factors such as age and BMI were found to drive the association with the H2FPEF score, we should point out a parallel involvement of cardiac remodeling and dysfunction. That is demonstrated by the observed independent associations between MVD measures and echocardiographic markers. We observed a significant inverse association between perfusion response to acetylcholine and LVMI in women, indicating that worse endothelial-dependent microvascular response is associated to worse cardiac remodeling [41]. Higher LVMI is also strongly related to the presence of coronary microvascular endothelial dysfunction, as impaired NO signaling favors cardiomyocyte hypertrophy [42]. The fact that elderly women with T2D are more prone to either develop LV hypertrophy in response to comorbidities, and are more susceptible to MVD than men, might partially explain this sex-specific association. Finally, we found a significant association between perfusion response to acetylcholine and LVGLS in the total population. LVGLS is a measure of longitudinal LV systolic function and is typically impaired in HFpEF and diabetic cardiomyopathy [42]. Lower LVGLS has also been independently associated with the burden of microvascular complications in subjects with T2D [43]. In this respect, our findings extend those of previous studies adding mechanistic information on the predominant role of endothelial-dependent mechanisms. However, perfusion responses were not associated with any echocardiographic components of the H2FPEF score such as E/E’ or with NT-proBNP. These negative finding might be related to a possible dissociation between systemic and cardiac involvement in early stages of HFpEF or LVDD, and to the weak relation that has been observed between peripheral and coronary MVD in HFpEF [33]. Nevertheless, peripheral endothelial dysfunction independently predicts HF hospitalizations in HFpEF patients [44], therefore holding an important prognostic value.

Impaired endothelial-dependent vasodilation might contribute to HFpEF risk through non-cardiac mechanisms, for instance by increasing systemic vascular resistance or activation of the endothelin system [45].

### The sex specific progression from LVDD to HFpEF

Mechanisms implicated in the progression from LVDD to HFpEF remain poorly understood and while the prevalence of LVDD is similar in men and women [16], the prevalence of HFpEF is approximately two-fold higher in women [36]. Comorbidities driving HFpEF have a higher impact on the risk of developing HFpEF in women [13, 15]. Because MVD is implicated in both T2D and HFpEF pathophysiology, mechanisms underlying MVD could represent a new (sex-specific) factor contributing to disease progression earlier and more substantially in women. Accordingly, in vivo indices of systemic MVD might represent novel markers of LVDD progression in women. However, the relationship between coronary and systemic MVD and between systemic and cardiac involvement in the different stages of HFpEF and LVDD remains to be further investigated in larger populations.

### Strengths and limitations

There are several limitations to this study. Firstly, since LASCA with combined iontophoresis is a novel technique, there is not yet a standardized and validated protocol available. This makes it difficult to generalize our findings, and to set ranges for normality. Secondly, 39 participants underwent LASCA approximately a year after echocardiography was performed, which may have affected our findings since cardiac functional measures can vary over time. However, we performed sensitivity analyses to address this issue, which did not substantially alter our findings. Thirdly, the assessment of most plasma biomarkers was not performed on the day of the visit, but rather during the annual diabetes check, approximately one year earlier. Therefore, plasma glucose concentrations were not measured in close proximity to the microvascular measurements and we could not account for them in our analyses. However, this does not hold for NT-proBNP plasma levels, demographics, blood pressure and anthropometrics, that were assessed on the day of the visit. Additionally, participants were not in a fasted state. Although they were inquired about the approximate time since their last meal and this variable was added as a covariate in our analyses, we cannot completely exclude that postprandial thermogenesis might have influenced cutaneous vasodilation, especially in response to insulin. Finally, we used a diagnostic tool such as the H2FPEF score, that was developed and validated in subjects with HF-like symptoms, while only a proportion of our population was symptomatic. Nevertheless, the H2FPEF score was also used in outpatients with cardiovascular risk factors, and associated to future HF [7], confirming a possible applicability to a broader group of patients. Moreover, we have carried out a sensitivity analysis including only participants showing symptoms and signs of HF. The lack of significance of associations in this group is likely to be attributed to the limited statistical power. Strengths of our study include the use of a novel, non-invasive and specific real-time method of assessing systemic microvascular function, that allowed us to investigate multiple mechanisms of endothelial-dependent and independent MVD.

## Conclusions

The present study showed that systemic endothelial dysfunction, detectable as impaired responses to insulin and acetylcholine, associates with increased risk of HFpEF in women with T2D. Further, impaired endothelium-dependent and -independent peripheral MVD associates with worse cardiac structure and function. Future studies are needed to test and validate our protocol in larger study populations and investigate prospectively the role of MVD in the development and progression of LVDD and HFpEF.

### Electronic supplementary material

Below is the link to the electronic supplementary material.

Supplementary Material 1

## Data Availability

The data used by the present study are available upon request.

## Abbreviations

HF: Heart failure
HFpEF: Heart failure with preserved ejection fraction
T2D type 2 diabetes. MVD: Microvascular dysfunction. NO:nitric oxide
cGMP: Cyclic guanosine monophosphate
LV: Left ventricular
LVDD: Left ventricular diastolic dysfunction
eNOS: NO synthase
DCS: Diabetes Care System cohort
LASCA: Laser speckle contrast analysis
SNP: Sodium nitroprusside
PASP: Pulmonary arterial pressure
AF: Atrial fibrillation
LVMI: LV mass index
LAVI: Left atrial volume index
GLS: Global longitudinal strain
LA: Left atrial
RV: Right ventricular
TAPSE: Tricuspid annular plane systolic excursion
HbA1c: Glycated hemoglobin
LDL-c: Low-density lipoprotein cholesterol
BMI: Body mass index
SBP: Systolic blood pressure
DBP: Diastolic blood pressure
CI: Confidence intervals

## References

[1] Savarese G, Lund LH (2017). Global Public Health Burden of Heart failure. Card Fail Rev.

[2] Vasan RS, Xanthakis V, Lyass A, Andersson C, Tsao C, Cheng S (2018). Epidemiology of left ventricular systolic dysfunction and heart failure in the Framingham Study: an echocardiographic study over 3 decades. JACC Cardiovasc Imaging.

[3] McDonagh TA, Metra M, Adamo M, Gardner RS, Baumbach A, Böhm M (2021). 2021 ESC Guidelines for the diagnosis and treatment of acute and chronic heart failure: developed by the Task Force for the diagnosis and treatment of acute and chronic heart failure of the European Society of Cardiology (ESC) with the special contribution of the heart failure Association (HFA) of the ESC. Eur Heart J.

[4] Shah SJ, Borlaug BA, Kitzman DW, McCulloch AD, Blaxall BC, Agarwal R (2020). Research Priorities for Heart failure with preserved ejection fraction: National Heart, Lung, and Blood Institute Working Group Summary. Circulation.

[5] Reddy YNV, Carter RE, Obokata M, Redfield MM, Borlaug BA (2018). A simple, evidence-based Approach to help Guide diagnosis of heart failure with preserved ejection fraction. Circulation.

[6] Sun Y, Wang N, Li X, Zhang Y, Yang J, Tse G (2021). Predictive value of H2 FPEF score in patients with heart failure with preserved ejection fraction. ESC Heart Fail.

[7] Suzuki S, Kaikita K, Yamamoto E, Sueta D, Yamamoto M, Ishii M (2020). H2 FPEF score for predicting future heart failure in stable outpatients with cardiovascular risk factors. ESC Heart Fail.

[8] Paulus WJ, Tschope C (2013). A novel paradigm for heart failure with preserved ejection fraction: comorbidities drive myocardial dysfunction and remodeling through coronary microvascular endothelial inflammation. J Am Coll Cardiol.

[9] Borlaug BA, Sharma K, Shah SJ, Ho JE (2023). Heart failure with preserved ejection fraction: JACC Scientific Statement. J Am Coll Cardiol.

[10] Borlaug BA, Jensen MD, Kitzman DW, Lam CSP, Obokata M, Rider OJ (2023). Obesity and heart failure with preserved ejection fraction: new insights and pathophysiological targets. Cardiovasc Res.

[11] Yang JH, Obokata M, Reddy YN, Redfield MM, Lerman A, Borlaug BA (2020). Endothelium-dependent and independent coronary microvascular dysfunction in patients with heart failure with preserved ejection fraction. Eur J Heart Fail.

[12] Masi S, Rizzoni D, Taddei S, Widmer RJ, Montezano AC, Luscher TF (2021). Assessment and pathophysiology of microvascular disease: recent progress and clinical implications. Eur Heart J.

[13] Beale AL, Meyer P, Marwick TH, Lam CS, Kaye DM (2018). Sex differences in cardiovascular pathophysiology: why women are overrepresented in heart failure with preserved ejection fraction. Circulation.

[14] Brown JM, Zhou W, Weber B, Divakaran S, Barrett L, Bibbo CF (2022). Low coronary flow relative to myocardial mass predicts heart failure in symptomatic hypertensive patients with no obstructive coronary artery disease. Eur Heart J.

[15] Kannel WB, Hjortland M, Castelli WP (1974). Role of diabetes in congestive heart failure: the Framingham study. Am J Cardiol.

[16] Groenewegen A, Rutten FH, Mosterd A, Hoes AW (2020). Epidemiology of heart failure. Eur J Heart Fail.

[17] Peterson LR, Herrero P, Schechtman KB, Racette SB, Waggoner AD, Kisrieva-Ware Z (2004). Effect of obesity and insulin resistance on myocardial substrate metabolism and efficiency in young women. Circulation.

[18] Riehle C, Abel ED (2016). Insulin signaling and heart failure. Circ Res.

[19] Prior JO, Quinones MJ, Hernandez-Pampaloni M, Facta AD, Schindler TH, Sayre JW (2005). Coronary circulatory dysfunction in insulin resistance, impaired glucose tolerance, and type 2 diabetes mellitus. Circulation.

[20] NHG-werkgroep, Bouma M, Dankers M, De Rooij A, Hart HE, Houweling ST, IJzerman RG, Janssen PGH, Kerssen A, Oud M, Palmen J, Van den Brink-Muinen A, Van den Donk M, Verburg-Oorthuizen AFE, Wiersma Tj. NHG-STANDAARD - Diabetes mellitus type 2 2018 [updated January 2023.

[21] Hellmann M, Roustit M, Cracowski J-L (2015). Skin microvascular endothelial function as a biomarker in cardiovascular diseases?. Pharmacol Rep.

[22] de Jongh RT, Serne EH, RG IJ, Jorstad HT, Stehouwer CD (2008). Impaired local microvascular vasodilatory effects of insulin and reduced skin microvascular vasomotion in obese women. Microvasc Res.

[23] Nagueh SF, Smiseth OA, Appleton CP, Byrd BF, Dokainish H, Edvardsen T (2016). Recommendations for the evaluation of left ventricular diastolic function by Echocardiography: an update from the American Society of Echocardiography and the European Association of Cardiovascular Imaging. Eur Heart J Cardiovasc Imaging.

[24] Reddy YN, Carter RE, Obokata M, Redfield MM, Borlaug BA (2018). A simple, evidence-based approach to help guide diagnosis of heart failure with preserved ejection fraction. Circulation.

[25] Sepehrvand N, Alemayehu W, Dyck GJB, Dyck JRB, Anderson T, Howlett J (2019). External validation of the H2F-PEF model in diagnosing patients with heart failure and preserved ejection fraction. Circulation.

[26] Lang RM, Badano LP, Mor-Avi V, Afilalo J, Armstrong A, Ernande L (2015). Recommendations for cardiac chamber quantification by echocardiography in adults: an update from the American Society of Echocardiography and the European Association of Cardiovascular Imaging. Eur Heart J Cardiovasc Imaging.

[27] van der Heijden AA, Rauh SP, Dekker JM, Beulens JW, Elders P, t Hart LM (2017). The Hoorn Diabetes Care System (DCS) cohort. A prospective cohort of persons with type 2 diabetes treated in primary care in the Netherlands. BMJ Open.

[28] Paulus WJ, Zile MR (2021). From systemic inflammation to myocardial fibrosis: the heart failure with preserved ejection Fraction paradigm revisited. Circ Res.

[29] Crea F, Bairey Merz CN, Beltrame JF, Kaski JC, Ogawa H, Ong P (2017). The parallel tales of microvascular angina and heart failure with preserved ejection fraction: a paradigm shift. Eur Heart J.

[30] Lee JF, Barrett-O’Keefe Z, Garten RS, Nelson AD, Ryan JJ, Nativi JN (2016). Evidence of microvascular dysfunction in heart failure with preserved ejection fraction. Heart.

[31] Lam CS, Arnott C, Beale AL, Chandramouli C, Hilfiker-Kleiner D, Kaye DM (2019). Sex differences in heart failure. Eur Heart J.

[32] Chandramouli C, Ting TW, Tromp J, Agarwal A, Svedlund S, Saraste A (2022). Sex differences in proteomic correlates of coronary microvascular dysfunction among patients with heart failure and preserved ejection fraction. Eur J Heart Fail.

[33] Shah SJ, Lam CS, Svedlund S, Saraste A, Hage C, Tan R-S (2018). Prevalence and correlates of coronary microvascular dysfunction in heart failure with preserved ejection fraction: PROMIS-HFpEF. Eur Heart J.

[34] Caballero AE, Arora S, Saouaf R, Lim SC, Smakowski P, Park JY (1999). Microvascular and macrovascular reactivity is reduced in subjects at risk for type 2 diabetes. Diabetes.

[35] Haas AV, Rosner BA, Kwong RY, Rao AD, Garg R, Di Carli MF (2019). Sex differences in coronary microvascular function in individuals with type 2 diabetes. Diabetes.

[36] Beale AL, Meyer P, Marwick TH, Lam CSP, Kaye DM (2018). Sex differences in Cardiovascular Pathophysiology: why women are overrepresented in heart failure with preserved ejection fraction. Circulation.

[37] Fisslthaler B, Benzing T, Busse R, Fleming I (2003). Insulin enhances the expression of the endothelial nitric oxide synthase in native endothelial cells: a dual role for akt and AP-1. Nitric Oxide.

[38] Peterson LR, Saeed IM, McGill JB, Herrero P, Schechtman KB, Gunawardena R (2012). Sex and type 2 diabetes: obesity-independent effects on left ventricular substrate metabolism and relaxation in humans. Obes (Silver Spring).

[39] Sabbah MS, Fayyaz AU, de Denus S, Felker GM, Borlaug BA, Dasari S (2020). Obese-inflammatory phenotypes in heart failure with preserved ejection fraction. Circ Heart Fail.

[40] Voges HK, Foster SR, Reynolds L, Parker BL, Devilee L, Quaife-Ryan GA (2023). Vascular cells improve functionality of human cardiac organoids. Cell Rep.

[41] Pieske B, Tschope C, de Boer RA, Fraser AG, Anker SD, Donal E (2020). How to diagnose heart failure with preserved ejection fraction: the HFA-PEFF diagnostic algorithm: a consensus recommendation from the heart failure Association (HFA) of the European Society of Cardiology (ESC). Eur J Heart Fail.

[42] Wang M, Li Y, Li S, Lv J (2022). Endothelial dysfunction and Diabetic Cardiomyopathy. Front Endocrinol (Lausanne).

[43] Pararajasingam G, Heinsen LJ, Larsson J, Andersen TR, Logstrup BB, Auscher S (2021). Diabetic microvascular complications are associated with reduced global longitudinal strain independent of atherosclerotic coronary artery disease in asymptomatic patients with diabetes mellitus: a cross-sectional study. BMC Cardiovasc Disord.

[44] Akiyama E, Sugiyama S, Matsuzawa Y, Konishi M, Suzuki H, Nozaki T (2012). Incremental prognostic significance of peripheral endothelial dysfunction in patients with heart failure with normal left ventricular ejection fraction. J Am Coll Cardiol.

[45] Hornstra JM, Serne EH, Eringa EC, Wijnker MC, de Boer MP, Yudkin JS (2013). Insulin’s microvascular vasodilatory effects are inversely related to peripheral vascular resistance in overweight, but insulin-sensitive subjects. Obes (Silver Spring).

