# Supplementary material for: Microvascular endothelial dysfunction in skin is associated with higher risk of heart failure with preserved ejection fraction in women with type 2 diabetes: the Hoorn Diabetes Care System Cohort
Source: Cardiovasc Diabetol. 2023 Sep 1;22:234. doi: 10.1186/s12933-023-01935-z (PMC10474683; doi:10.1186/s12933-023-01935-z)
Supplement: Supplementary file 1 — Supplementary Material 1 [file 12933_2023_1935_MOESM1_ESM.docx]

**Impaired microvascular endothelial function in skin is associated with higher risk of heart failure with preserved ejection fraction in women with type 2 diabetes from the Hoorn Diabetes Care System Cohort**

E. Dal Canto, L. van Deursen, A. G. Hoek, P. J. M. Elders, H. M. den Ruijter, J. van der Velden, V. van Empel, E.H. Serné, E. C. Eringa, J.W.J Beulens.

**Additional material**

**Appendix**

**Assessment of microvascular function**

Microvascular function was evaluated in the skin using semi-quantitative LASCA technology of the PeriCam PSI system (Perimed instruments, Järfälla-Stockholm, Sweden). The PeriCam PSI System is a blood perfusion imager that allow to visualize tissue blood perfusion in real time by using an invisible near infra-red laser (785 nm). The beam is spread over the measurement area by a diffuser, creating a speckle pattern. It was combined with iontophoresis of vasoactive substances: insulin (1%), acetylcholine (1%) and sodium nitroprusside (SNP, 0.1%) (1).

Two protocols were carried out for each participant. During the first, insulin and NaCl (0.9%) were used (2). Two iontophoresis chambers with administration areas of 1.54 cm^2^ attached to the ventral surface of the forearm using adhesive discs, were used to deliver the substances to the skin, avoiding hair, broken skin, or areas of increased skin pigmentation as much as possible. Dispersive electrodes were attached to the ventral surface of the wrist at approximately 15 cm distance from the drug delivery electrodes to complete the electrical current circuit. During the first protocol the dominant arm was used and a total dose of 360,00 µAmin was delivered for iontophoresis of insulin, in 20 µAmin per minute lasting for 18 minutes (27). For the second protocol, the other forearm was used, and acetylcholine and SNP were delivered to the skin. Acetylcholine was delivered as described previously (3) in 7 pulses of 233,33 µAmin with a negative drug polarity, lasting 20 seconds followed by a 40 second pause, for a total of 8 minutes. SNP was delivered in 9 pulses of 600,00 µAmin with a positive drug polarity, lasting 20 seconds followed by a 60 second pause for a total of 14 minutes and 30 seconds. For both protocols, the working distance between the laser head and the surface of the forearm was set at approximately 15-20 cm. The laser measurement area and image acquisition rate were set at 100cm^2^ and 21 images per second, respectively. A dedicated software (PimSoft version 1.6) was used to assess the baseline perfusion, the perfusion plateau induced by the delivery of each substance, the maximum absolute and relative change in perfusion due to the substance (perfusion response, PR) calculated as 100% x ([Perfusion plateau – Baseline flow]/Baseline flow). Skin perfusion outside the area of interest was used as control.

The time from baseline to the reach of the plateau in perfusion was determined by a trained investigator by manually locating the onset of the plateaus on the perfusion curves.

**Additional Table 1**. Measures of systemic microvascular function stratified by sex.

|  | Men | Women | P value |
| --- | --- | --- | --- |
| Average baseline perfusion, PU | 29.7 (27.0-35.0) | 35.0 (28.4-39.3) | **0.001** |
| Insulin | **(n=76)** | **(n=77)** |  |
| Perfusion plateau, PU | 56.5 (44.0-66.5) | 67.0 (46.0-84.5) | **0.043** |
| Absolute change, % | 24.5 (58.8-71.8) | 32.9 (10.3-57.1) | 0.234 |
| Relative change, % | 87.0 (38.5-129.8) | 109.2 (31.1-173.7) | 0.427 |
| Time to plateau, min | 14.27 (14.15 -17.43) | 16.25 (14.12-18.36) | **0.011** |
| Acetylcholine | **(n=76)** | **(n=76)** |  |
| Perfusion plateau, PU | 90.8 (74.2-104.6) | 99.2 (88.1-112.9) | **0.001** |
| Absolute change, % | 58.8 (45.0-71.8) | 63.2 (51.3-75.9) | 0.058 |
| Relative change, % | 195.9 (149.2-240.6) | 193.0 (135.9-242.2) | 0.635 |
| Time to plateau, min | 7.26 (7.12-8.44) | 6.27 (6.10-8.41) | 0.594 |
| SNP | **(n=76)** | **(n=76)** |  |
| Perfusion plateau, PU | 83.0 (73.7-97.9) | 87.1 (73.6-104.9) | 0.104 |
| Absolute change, % | 51.9 (40.7-65.2) | 49.6 (35.1-67.9) | 0.839 |
| Relative change, % | 176.8 (139.0-220.9) | 145.3 (89.5-216.1) | 0.055 |
| Time to plateau, min | 11.12 (10.27-13.45) | 12.15 (9.25-14.40) | 0.199 |

Results are presented as median (IQR range) and compared using a Wilcoxon Rank Sum test.

SNP = sodium nitroprusside, PU = perfusion units.

**Additional Table 2.** Cardiac function and structure measures of study participants stratified by sex

|  | Men, n=77 | Women n=77 | Men, n=77 |
| --- | --- | --- | --- |
| Left ventricular geometry | | | |
| LVMI, g/m2 | 100.2 ± 20.6 | 82.5 ± 18.4 | **<0.001** |
| LVEDV, ml | 132.1 ± 24.6 | 96.2 ± 21.0 | **<0.001** |
| RWT | 0.40 ± 0.05 | 0.4 ± 0.05 | 0.376 |
| LV geometry, n (%) |  |  | **0.010** |
| Normal geometry | 44 (57.9) | 45 (59.2) |  |
| Concentric remodeling | 15 (19.7) | 17 (22.4) |  |
| Concentric hypertrophy | 0 | 7 (9.2) |  |
| Eccentric hypertrophy | 17 (22.4) | 7 (9.2) |  |
| Left ventricular systolic function | | | |
| LVEF biplane, % | 59.1 ± 5.5 | 59.0 ± 4.6 | 0.897 |
| LV global strain, % | -17.1 ± 1.9 | -16.8 ± 2.5 | 0.332 |
| Stroke volume, mL | 84.2 ± 18.7 | 67.8 ± 19.9 | **<0.001** |
| Left ventricular diastolic function and atrial structure and function | | | |
| E wave, cm/s | 67.1 ± 14.4 | 72.0 ± 18.0 | 0.066 |
| Deceleration time, ms | 198.8 ± 30.9 | 197.9 ± 31.0 | 0.855 |
| E’ medial, m/s | 5.6  ± 1.3 | 5.1 ± 1.1 | **0.019** |
| E’ lateral, m/s | 6.8 ± 1.7 | 6.3 ± 1.5 | 0.053 |
| E’ mean, cm/s | 6.2 ± 1.4 | 5.7 ± 1.2 | **0.017** |
| E/E’, mean | 11.4 ± 3.0 | 13.3 ± 4.3 | **0.002** |
| Presence of LVDD^1^, n (%) |  |  | 0.550 |
| Normal diastolic function, | 35 (67.3) | 32 (57.1) |  |
| Indeterminate diastolic function | 13 (25) | 18 (32.1) |  |
| Impaired diastolic function | 4 (7.7) | 6 (10.7) |  |
| LAVI max, mL/m2 | 29.0 ± 7.8 | 28.5 ± 9.6 | 0.706 |
| A’ mean, cm/s | 9.3 ± 1.3 | 8.8 ± 1.6 | **0.037** |
| LA global strain, % | -29.6 ± 7.0 | -26.7 ± 6.5 | **0.025** |
| Right ventricular and right atrial structure and function | | | |
| RV fractional area shortening, % | 42.7 ± 10.0 | 45.7 ± 8.2 | 0.051 |
| TAPSE, mm | 24.9 ± 3.5 | 22.6 ± 3.0 | **<0.001** |
| RV end-diastolic area, cm^2^ | 22.7 ± 4.8 | 17.1 ± 3.8 | **<0.001** |
| Right atrial volume max, mL | 39.6 ± 23.4 | 30.6 ± 14.6 | **0.006** |
| Tricuspid regurgitation velocity, m/s | 2.4 ± 0.3 | 2.3 ± 0.2 | 0.424 |
| RV S’, cm/s | 10.6 ± 2.4 | 9.8 ± 2.1 | 0.033 |
| H2FPEF score^2^, % | 23.0 ± 14.6 | 30.0 ± 19.5 | **0.014** |

Results are presented as mean ± standard deviation or frequencies (percentages) and compared between men and women using t-test or chi-square tests, where appropriate.

^1^Presence of LVDD is defined according to the Nagueh classification of left ventricular diastolic dysfunction (4).

^2^The continuous H2FPEF score is an estimation of the probability of developing HFpEF, based on the formula developed by Reddy et al (5).

LVMI = left ventricular mass index, LVEVD = left ventricular end diastolic volume, RWT = relative wall thickness, LVEF = left ventricular ejection fraction, LAVI = left atrial volume index, RV=right ventricular, TAPSE = tricuspid annular plane systolic excursion, LV = left ventricular, LA = left atrial.

**Additional Table 3**. Associations between log-transformed relative change in perfusion from baseline to plateau for each substance and single parameters of LVDD and HFPEF.

|  | E’ mean  β (95% CI) | LVMI  β (95% CI) | LAVI  β (95% CI) | LV Strain  β (95% CI) | LA strain  β (95% CI) | TAPSE  β (95% CI) | NT-proBNP  β (95% CI) | SBP  β (95% CI) |
| --- | --- | --- | --- | --- | --- | --- | --- | --- |
| Relative change in perfusion Insulin | | |  | | |  | | |
| Total population | | |  | | |  | | |
| Model 1 | -0.6 (-2.7; 1.6) | 9.1 (-21.7; 40.0) | 0.8 (-11.7; 13.3) | 1.8 (-1.6; 5.2) | 4.4 (-8.6; 17.3) | 1.0 (-4.7; 6.6) | -226.1 (-600.0; 147.8) | -1.5 (-27.6; 24.5) |
| Model 2 | -0.9 (-3.0; 1.3) | 8.0 (-23.1; 39.1) | 0.1 (-12.5; 12.6) | 1.5 (-2.0; 4.9) | 4.1 (-9.2; 17.4) | 1.0 (-4.8; 6.6) | -198.7 (-581.6; 184.2) | -0.8 (-27.2; 25.7) |
| Men | | | | | | | | |
| Model 1 | -1.2 (-5.0; 2.6) | -2.1 (-54.6; 50.4) | 0.3 (-20.2, 20.7) | 2.0 (-2.1; 6.2) | -5.6 (-29.3; 18.1) | 7.7 (-1.3; 16.6) | -63.6 (-364.6; 237.4) | -30.2 (-70.8; 10.4) |
| Model 2 | -1.7 (-5.5; 2.1) | -1.6 (-55.8; 52.5) | -2.5 (-23.1; 18.0) | 1.5 (-2.6; 5.6) | -6.3 (-31.1; 18.5) | 7.5 (-1.6; 16.7) | -83.4 (-390.3; 223.5) | -28.5 (-70.7; 13.8) |
| Women | | | | | | | | |
| Model 1 | -0.5 (-3.2; 2.3) | 31.8 (-9.6; 73.3) | 0.9 (-16.3; 18.2) | 2.8 (-2.5; 8.1) | 4.6 (-12.4; 21.6) | -1.9 (-9.5; 5.6) | -389.5 (-1031.2; 252.2) | 3.4 (-30.9; 37.8) |
| Model 2 | -0.9 (-3.7; 2.0) | 32.2 (-11.0; 75.4) | 0.8 (-17.5; 19.0) | 2.8 (-2.8; 8.4) | 3.1 (-14.7; 20.9) | -3.1 (-10.7; 4.6) | -309.3 (-980.7; 362.1) | 2.4 (-33.0; 37.9) |
| Relative change in perfusion Ach | | | | | | | | |
| Total population | | | | | | | | |
| Model 1 | 0.9 (-1.1; 2.9) | -10.3 (-39.0; 18.3) | -3.1 (-14.6; 8.6) | **3.3 (0.2; 6.4)*** | -2.5 (-14.6; 9.5) | 1.5 (-3.6; 6.7) | -207.1 (-568.3; 154.1) | -16.7 (-40.7; 7.4) |
| Model 2 | 1.0 (-1.1; 3.1) | -20.7 (-50.9; 9.5) | -4.3 (-16.5; 8.0) | **3.3 (0.0; 6.6)*** | -3.2 (-16.1; 9.7) | 0.2 (-5.2; 5.7) | -190.8 (-571.2; 189.6) | -12.0 (-37.7; 13.8) |
| Men | | | | | | | | |
| Model 1 | 1.9 (-1.9; 5.7) | -0.9 (-54.7; 53.0) | 6.8 (-14.0; 27.7) | 2.5 (-1.7; 6.7) | -0.7 (-24.8; 23.3) | 3.0 (-6.2; 12.2) | -216.2 (-516.5; 84.1) | -10.3 (-53.4; 32.9) |
| Model 2 | 1.0 (-3.1; 5.1) | -1.7 (-60.5; 57.2) | -0.5 (-22.7; 21.7) | 1.6 (-2.9; 6.0) | -2.3 (-29.0; 24.4) | 1.8 (-8.0; 11.7) | -283.9 (-606.8; 39.1) | -3.0 (-50.2; 44.3) |
| Women | | | | | | | | |
| Model 1 | 1.7 (-1.1; 4.5) | -35.6 (-78.3; 7.1) | -1.0 (-19.2; 17.2) | 3.9 (-1.8; 9.7) | -3.6 (-21.4; 14.1) | -0.2 (-7.5; 8.0) | -248.3 (-960.7; 464.2) | -32.5 (-68.1; 3.1) |
| Model 2 | 2.5 (-0.6; 5.5) | -**59.5 (-104.3; -14.6)**** | 0.7 (-19.5; 20.8) | 3.9 (-2.6; 10.4) | -5.8 (-25.1; 13.5) | -3.6 (-11.8; 4.6) | -145.4 (-918.4; 627.6) | -20.7 (-60.1; 18.8) |
| Relative change in perfusion SNP | | | | | | | | |
| Total population | | | | | | | | |
| Model 1 | -0.0 (-0.6; 0.5) | -1.3 (-9.4; 6.8) | -0.0 (-3.3; 3.2) | -0.1 (-1.0; 0.8) | -0.7 (-4.1; 2.7) | -0.5 (-1.9; 1.0) | 6.3 (-95.2; 107.8) | 0.9 (-6.0; 7.8) |
| Model 2 | 0.0 (-0.5; 0.6) | -0.8 (-9.0; 7.5) | -0.3 (-3.6; 3.0) | 0.0 (-0.9; 0.9) | -0.4 (-4.0; 3.1) | -0.5 (-2.0; 1.0) | 2.3 (-103.3; 107.9) | 0.7 (-6.3; 7.7) |
| Men | | | | | | | | |
| Model 1 | -1.5 (-5.9; 2.8) | 31.6 (-29.3; 92.5) | -3.4 (-27.2; 20.3) | 1.7 (-3.1; 6.5) | -9.5 (-37.3; 18.2) | -1.4 (-11.9; 9.0) | **-346.7 (-678.9; -14.4)*** | **-50.6 (-95.5; -5.7)*** |
| Model 2 | -3.0 (-7.4; 1.4) | 38.3 (-26.7; 103.3) | -10.7 (-35.2; 13.8) | 0.8 (-4.1; 5.7) | -10.1 (-40.2; 19.9) | -1.7 (-12.8; 9.3) | **-386.1 (-721.8; -14.4)*** | -49.0 (-98.3; 0.2) |
| Women | | | | | | | | |
| Model 1 | -0.1 (-0.7; 0.5) | -2.7 (-13.2; 7.9) | -0.2 (-4.2; 3.8) | -0.2 (-1.5; 1.1) | -0.9 (-4.7; 3.0) | -0.1 (-1.8; 1.7) | 42.4 (-109.1; 193.9) | 0.1 (-8.3; 8.5) |
| Model 2 | -0.1 (-0.7; 0.6) | -1.6 (-12.5; 9.3) | -0.4 (-4.7; 3.8) | -0.1 (-1.5; 1.3) | -0.1 (-4.1; 3.9) | 0.3 (-1.5; 2.0) | 26.5 (-131.5; 184.5) | -1.1 (-9.8; 7.7) |

* P < 0.05 level.** p<0.01 level.

The determinants were log-transformed before they were entered into this model. N=151, with 75 men and 76 women. Only for PASP more than 5% of values were missing, with n= 108, with 52 men and 56 women. Missing cases were excluded pair-wise.

Model 1 adjusts for sex, age, BMI, HbA1c, LDL cholesterol, presence of hypertension, serum creatinine, albumin-creatinine ratio, diabetes duration, smoking status, prior CVD, NTproBNP value and the relative change in perfusion of the skin outside the measurement area during the protocol. For the regression analyses with relative change in perfusion due to insulin as determinant, model 1 additionally adjusts for change in perfusion due to delivery of NaCl and time since last meal. Model 2 additionally adjusts for metformin use, insulin use, and use of anti-hypertensive medication.

LVMI = left ventricular mass index, LAVI = left atrial volume index, LV= left ventricular, LA left atrial, TAPSE = tricuspid annular plane systolic excursion, SBP = systolic blood pressure.

**Additional Table 4** Sensitivity analyses for the associations between log-transformed relative change in perfusion from baseline to plateau for each substance and H2FPEF score^1^ and each of the components of the score in the total study population and in men and women separately

|  | Total population  B (95% CI) | | Men  B (95% CI) | | Women  B (95% CI) |
| --- | --- | --- | --- | --- | --- |
| Relative change in perfusion insulin | | | | | |
| *Excluded if a perfusion plateau was not observed* | | | | | |
| H2FPEF score | **-37.6 (-64.7; -10.4)**** | | -22.9 (-64.5; 18.6) | | **-55.9 (-94.6; -17.1)**** |
| Age | **-14.0 (-24.6; -3.4)**** | | -16.9 (-36.4; 2.5) | | -12.2 (-25.2; 0.8) |
| BMI | **-11.3 (-19.7; -2.9)**** | | -8.9 (-21.0; 3.3) | | **-15.6 (-28.9; -2.2)*** |
| AF | 0.1 (-0.1; 0.3) | | 0.2 (-0.1; 0.5) | | -0.1 (-0.3; 0.2) |
| E/E’ | -1.8 (-9.2; 5.6) | | 3.2 (-6.0; 12.3) | | -4.2 (-15.5; 7.2) |
| PASP | -5.5 (-15.4; 4.5) | | -4.2 (-24.1; 15.7) | | -5.0 (-17.8; 7.8) |
| *Excluded if microvascular assessment took place >6 months after the echo* | | | | | |
| H2FPEF score | **-38.8 (-70.9; -6.7)*** | | -9.6 (-51.5; 32.3) | | **-70.5 (-132.2; -8.7)*** |
| Age | **-17.3 (-29.3; -5.2)**** | | -15.8 (-33.7; 2.2) | | -8.4 (-23.0; 6.3) |
| BMI | **-12.6 (-22.6; -2.5)*** | | -6.5 (-18.7; 5.7) | | -11.3 (-32.6; 9.9) |
| AF | 0.1 (-0.1; 0.4) | | 0.3 (-0.1; 0.1) | | -0.0 (-0.6; 0.5) |
| E/e’ | 0.2 (-6.1; 6.4) | | 1.2 (-7.0; 9.5) | | **-**3.1 (-15.7; 9.5) |
| PASP | -1.4 (-14.1; 11.3) | | -4.7 (-25.0; 15.7) | | 0.6 (-25.7; 26.8) |
| *Excluded if asymptomatic* | | | | | |
| H2FPEF score | -30.3 (-98.7; 38.1) | |  | |  |
| Age | -18.8 (-37.8; 0.2) | |  | |  |
| BMI | -8.7 (-31.9; 14.5) | |  | |  |
| AF | 0.4 (-0.3; 1.0) | |  | |  |
| E/e’ | -8.8 (-30.8; 19.1) | |  | |  |
| PASP | -17.9 (not available) | |  | |  |
| Relative change in perfusion acetylcholine | | | | | |
| *Excluded if microvascular assessment took place >6 months after the echo* | | | | | |
| H2FPEF score | -35.5 (-74.0; 3.0) | | -14.2 (-60.7; 32.2) | | **-45.8 (-104.0; -12.4)**** |
| Age | **-27.0 (-40.6; -13.4)**** | | **-33.0 (-51.8; -14.1)**** | | -14.7 (-30.1; 0.6) |
| BMI | -4.6 (-17.7; 8.5) | | -2.8 (-17.9; 12.3) | | -1.5 (-23.2; 20.2) |
| AF | 0.0 (-0.3; 0.3) | | -0.1 (-0.5; 0.3) | | -0.1 (-0.4; 0.3) |
| E/E’ | -0.2 (-7.7; 7.3) | | 5.4 (-4.1; 14.9) | | -4.9 (-17.9; 8.2) |
| PASP | -14.7 (-30.0; 3.0) | | -7.5 (-31.2; 16.0) | | -2.5 (-30.6; 25.6) |
| *Excluded if asymptomatic* | | | | | |
| H2FPEF score | **-**32.8 (-90.3; 24.7) | |  | |  |
| Age | -6.5 (-23.3; 10.3) | |  | |  |
| BMI | -12.3 (-29.0; 4.3) | |  | |  |
| AF | 0.2 (-0.4; 0.8) | |  | |  |
| E/e’ | 0.9 (-16.5; 18.3) | |  | |  |
| PASP | -3.2 (-20.5; 14.2) | |  | |  |
| Relative change in perfusion SNP | | | | | |
| *Excluded if microvascular assessment took place >6 months after the echo* | | | | | |
| H2FPEF score | -3.3 (-12.4; 5.8) | | -36.6 (-87.7; 14.6) | | -1.9 (-14.2; 10.4) |
| Age | **-3.6 (-6.9; -0.2)*** | | **-24.6 (-48.5; -0.7)*** | | -2.0 (-4.7; 0.7) |
| BMI | 0.2 (-2.6; 3.0) | | -8.4 (-23.8; 7.0) | | 1.2 (-2.4; 4.7) |
| AF | -0.0 (-0.1; 0.1) | | 0.1 (-0.3; 0.6) | | 0.0 (-0.1; 0.1) |
| E/E’ | -0.0 (-1.7; 1.6) | | 0.9 (-9.5; 11.3) | | 0.9 (-1.4; 3.1) |
| PASP | -1.1 (-4.4; 2.1) | | **-25.0 (-48.7; -1.3)*** | | -0.5 (-4.8; 3.8) |
| *Excluded if asymptomatic* | | | | | |
| H2FPEF score | | -26.0 (-82.1; 30.1) | |  |  |
| Age | | -7.2 (-24.2; 9.8) | |  |  |
| BMI | | -5.9 (-24.2; 12.4) | |  |  |
| AF | | 0.2 (-0.3; 0.6) | |  |  |
| E/e’ | | -4.4 (-21.7; 12.9) | |  |  |
| PASP | | -8.2 (NA) | |  |  |

* P < 0.05 level.** p<0.01 level. The determinants (relative change in perfusion from baseline to plateau for each of the substances) were first log-transformed and then added to the model. The results of the fully adjusted model (model 2) are presented. This model adjusts for age, sex, BMI, Hba1c, LDL cholesterol, presence of hypertension, serum creatinine, albumin-creatinine ratio, diabetes duration, smoking status, prior CVD, NTproBNP value, metformin use, insulin use, use of anti-hypertensive medication, and the relative change in perfusion of the skin outside the measurement area during the protocol. The models with relative change in perfusion due to insulin as determinant additionally adjust for the time since last meal and the relative change in perfusion due to NaCl.

^1^The continuous H2FPEF score is an estimation of the probability of developing HFpEF, based on the formula developed by Reddy et al. (5).

BMI = body mass index, AF = atrial fibrillation, PASP = pulmonary arterial systolic pressure, SNP=sodium nitroprusside.

**Additional Table 5**. Sensitivity analyses for the associations between log-transformed relative change in perfusion from baseline to plateau for each substance and single parameters of LVDD and HFPEF.

|  | E’ mean  β (95% CI) | LVMI  β (95% CI) | LAVI  β (95% CI) | LV Strain  β (95% CI) | LA strain  β (95% CI) | TAPSE  β (95% CI) | NT-proBNP  β (95% CI) | SBP  β (95% CI) |
| --- | --- | --- | --- | --- | --- | --- | --- | --- |
| Relative change in perfusion Insulin | | |  | | |  | | |
| *Excluded if a perfusion plateau was not observed* | | |  | | |  | | |
| Total population | -2.0 (-4.5; 0.5) | **40.1 (11.3; 69.0)**** | -4.4 (-18.7; 10.0) | 2.2 (-2.0; 6.5) | 10.2 (-4.7; 25.2) | 3.4 (-3.3; 10.1) | -259.2 (-722.1; 203.7) | 7.72 (-21.1; 36.6) |
| Men | -2.4 (-6.8; 2.1) | 23.1 (-30.7; 76.8) | -9.7 (-35.8; 16.4) | 0.4 (-4.9; 5.8) | -0.2 (-28.4; 28.0) | **11.4 (0.1; 22.8)*** | -144.1 (-507.6; 219.3) | -17.1 (-64.5; 30.4) |
| Women | -2.2 (-5.4; 1.0) | **62.2 (26.2; 98.2)**** | 0.4 (-19.4; 20.1) | 3.6 (-3.4; 10.7) | 4.8 (-15.7; 25.4) | -0.9 (-10.1; 8.4) | -271.0 (-1108.7; 566.7) | 11.7 (-23.6; 47.0) |
| *Excluded if microvascular assessment took place >6 months after the echo* | | | | | | | | |
| Total population | -0.7 (-3.7; 2.2) | 8.3 (-34.6; 51.1) | 0.4 (-14.9; 15.8) | 0.2 (-3.1; 3.4) | 6.0 (-12.6; 24.6) | 5.1 (-3.0; 13.1) | -165.4 (-766.4; 435.5) | 8.0 (-30.1; 46.2) |
| Men | 0.2 (-4.3; 4.8) | -5.7 (-68.0; 56.6) | -10.2 (-32.4; 12.1) | 2.9 (-1.7; 7.5) | -4.8 (-34.9; 25.3) | 10.5 (-0.5; 21.6) | -62.6 (-254.6; 129.4) | -28.6 (-79.0; 21.8) |
| Women | 0.3 (-4.6; 4.0) | 43.5 (-36.1; 123.2) | 6.8 (-16.3; 30.0) | -1.0 (-6.5; 4.4) | 14.4 (-20.8; 49.6) | 3.4 (-8.8; 15.5) | -812.3 (-2273.7; 649.0) | 1.0 (-66.5; 68.5) |
| *Excluded if asymptomatic* | | | | | | | | |
| Total population | 1.2 (-2.9; 5.3) | -18.6 (-93.5; 56.4) | 6.3 (-31.2; 43.9) | 6.1 (-1.3; 13.4) | 8.0 (-36.4; 52.5) | -8.2 (-23.8; 7.4) | -268.2 (-888.6; 352.3) | 45.1 (-26.5; 116.7) |
| Relative change in perfusion Ach | | |  | | |  | | |
| *Excluded if microvascular assessment took place >6 months after the echo* | | | | | | | | |
| Total population | 0.8 (-2.8; 4.3) | -49.7 (-101.2; 1.8) | -3.9 (-22.4; 14.6) | -1.4 (-5.2; 2.4) | -10.9 (-33.3; 11.6) | 2.6 (-6.8; 12.1) | -192.5 (-956.9; 571.8) | -24.1 (-71.1; 22.8) |
| Men | -1.9 (-7.2; 3.4) | 15.5 (-61.2; 92.2) | -12.4 (-38.6; 13.8) | -1.6 (-7.3; 4.0) | -12.7 (-47.5; 22.2) | 1.1 (-12.6; 14.9) | -92.2 (-359.2; 174.8) | 20.6 (-41.7; 83.0) |
| Women^1^ | 0.2 (-5.1; 4.8) | -34.8 (-110.5; 40.9) | 6.2 (-21.4; 33.8) | 0.8 (-5.6; 7.2) | -1.3 (-45.1; 42.5) | 7.0 (-1.8; 16.9) | 291.4 (-337.4; 920.1) | 29.7 (-34.2; 93.6) |
| *Excluded if asymptomatic* | | | | | | | | |
| Total population | 2.6 (-0.9; 6.0) | -17.6 (-76.7; 41.4) | 7.0 (-21.6; 35.7) | 1.0 (-6.7; 8.7) | -11.7 (-45.9; 22.6) | 4.1 (-7.9; 16.2) | -142.6 (-702.3; 417.0) | -7.0 (-67.9; 53.8) |
| Relative change in perfusion SNP | | |  | | |  | | |
| *Excluded if microvascular assessment took place >6 months after the echo* | | | | | | | | |
| Total population | -0.0 (-0.8; 0.8) | -4.1 (-15.5; 7.3) | -1.3 (-5.3; 2.8) | 0.5 (-0.4; 1.3) | 0.7 (-4.2; 5.6) | -0.4 (-2.5; 1.7) | 50.0 (-121.4; 221.4) | -0.1 (-10.3; 10.1) |
| Men | -4.1 (-9.6; 1.4) | 61.8 (-18.1; 141.7) | 0.9 (-27.3; 29.1) | -0.6 (-6.3; 5.1) | -29.0 (-67.1; 9.0) | -6.7 (-20.8; 7.6) | -150.8 (-416.6; 115.1) | -36.4 (-102.8; 30.0) |
| Women | -0.1 (-0.9; 0.8) | -5.0 (-19.6; 9.7) | -0.5 (-5.0; 4.1) | -0.2 (-1.3; 0.9) | -0.2 (-7.0; 6.7) | 1.0 (-1.3; 3.4) | 83.9 (-216.3; 384.1) | 0.7 (-11.3; 12.6) |
| *Excluded if asymptomatic* | | | | | | | | |
| Total population | 2.3 (-1.2; 5.7) | 4.4 (-52.4; 61.3) | 1.6 (-27.4; 30.5) | 1.0 (-6.2; 8.1) | -1.1 (-39.1; 36.8) | -6.8 (-18.4; 4.9) | 91.8 (-385.3; 569.0) | 0.2 (-59.1; 59.5) |

* P < 0.05 level.** p<0.01 level.

The determinants were log-transformed before they were entered into this model. N=151, with 75 men and 76 women. Only for PASP more than 5% of values were missing, with n= 108, with 52 men and 56 women. Missing cases were excluded pair-wise.

The results of the final regression model are shown. This model adjusts for age, sex, BMI, Hba1c, LDL cholesterol, presence of hypertension, serum creatinine, diabetes duration, smoking status, prior CVD, NTproBNP value, metformin use, insulin use, use of anti-hypertensive medication, and the relative change in perfusion of the skin outside the measurement area during the protocol. The models with relative change in perfusion due to insulin as determinant additionally adjust for the time since last meal and the relative change in perfusion due to NaCl.

^12^Listwise exclusion of missing values for this row, because of impossible tolerances for pairwise exclusion

LVMI = left ventricular mass index, LAVI = left atrial volume index, LV= left ventricular, LA left atrial, TAPSE = tricuspid annular plane systolic excursion, SBP = systolic blood pressure.

**References**

1. de Jongh RT, Serne EH, RG IJ, Jorstad HT, Stehouwer CD. Impaired local microvascular vasodilatory effects of insulin and reduced skin microvascular vasomotion in obese women. Microvasc Res. 2008;75(2):256-62.

2. La Fountaine MF, Rivera DR, Radulovic M, Bauman WA. The hemodynamic actions of insulin are blunted in the sublesional microvasculature of healthy persons with spinal cord injury. Am J Phys Med Rehabil. 2013;92(2):127-35.

3. Emanuel AL, Nieuwenhoff MD, Klaassen ES, Verma A, Kramer MH, Strijers R, et al. Relationships Between Type 2 Diabetes, Neuropathy, and Microvascular Dysfunction: Evidence From Patients With Cryptogenic Axonal Polyneuropathy. Diabetes Care. 2017;40(4):583-90.

4. Nagueh SF, Smiseth OA, Appleton CP, Byrd BF, 3rd, Dokainish H, Edvardsen T, et al. Recommendations for the Evaluation of Left Ventricular Diastolic Function by Echocardiography: An Update from the American Society of Echocardiography and the European Association of Cardiovascular Imaging. Eur Heart J Cardiovasc Imaging. 2016;17(12):1321-60.

5. Reddy YN, Carter RE, Obokata M, Redfield MM, Borlaug BA. A simple, evidence-based approach to help guide diagnosis of heart failure with preserved ejection fraction. Circulation. 2018;138(9):861-70.
